# Improved DNA Vaccine Delivery with Needle-Free Injection Systems

**DOI:** 10.3390/vaccines11020280

**Published:** 2023-01-28

**Authors:** Carmen Ledesma-Feliciano, Ros Chapman, Jay W. Hooper, Kira Elma, Darin Zehrung, Miles B. Brennan, Erin K. Spiegel

**Affiliations:** 1 PharmaJet, 400 Corporate Circle, Unit N, Golden, CO 80401, USA; 2 Institute of Infectious Disease and Molecular Medicine, Faculty of Health Science, University of Cape Town, Cape Town 7925, South Africa; 3 Division of Medical Virology, Department of Pathology, University of Cape Town, Cape Town 7925, South Africa; 4 Molecular Virology Branch, Virology Division, United States Army Medical Research Institute of Infectious Diseases, Fort Detrick, Frederick, MD 21702, USA; 5 ZabBio, Inc., 11760 Sorrento Valley Road, San Diego, CA 92121, USA

**Keywords:** needle-free injection, jet injection, DNA, DNA vaccines, viral vaccines, electroporation, gene gun, particle-mediated epidermal delivery, pandemic viruses, emerging infectious diseases

## Abstract

DNA vaccines have inherent advantages compared to other vaccine types, including safety, rapid design and construction, ease and speed to manufacture, and thermostability. However, a major drawback of candidate DNA vaccines delivered by needle and syringe is the poor immunogenicity associated with inefficient cellular uptake of the DNA. This uptake is essential because the target vaccine antigen is produced within cells and then presented to the immune system. Multiple techniques have been employed to boost the immunogenicity and protective efficacy of DNA vaccines, including physical delivery methods, molecular and traditional adjuvants, and genetic sequence enhancements. Needle-free injection systems (NFIS) are an attractive alternative due to the induction of potent immunogenicity, enhanced protective efficacy, and elimination of needles. These advantages led to a milestone achievement in the field with the approval for Restricted Use in Emergency Situation of a DNA vaccine against COVID-19, delivered exclusively with NFIS. In this review, we discuss physical delivery methods for DNA vaccines with an emphasis on commercially available NFIS and their resulting safety, immunogenic effectiveness, and protective efficacy. As is discussed, prophylactic DNA vaccines delivered by NFIS tend to induce non-inferior immunogenicity to electroporation and enhanced responses compared to needle and syringe.

## 1. DNA as a Platform for Prophylactic Vaccine Development

DNA vaccines have successfully emerged as a viable and compelling alternative to other types of vaccines targeted for human use, including inactivated virus, live attenuated virus, subunit proteins, virus-like particles (VLP), virally vectored recombinant, and even closely related mRNA vaccines. The advantages of DNA vaccines compared to other types of vaccines include an improved safety profile, ease and speed of development by leveraging the power of genomics, and potentially enhanced cost-effectiveness due to a more simplified production process [1,2,3,4]. This allows the ability to quickly and more safely manufacture vaccines at scale given the challenges of the lengthy and potentially hazardous process of working with live pathogens to produce proteins or inactivated viruses. DNA vaccines are stable at ambient temperatures, thus eliminating the ultra-low-temperature storage and cold chain transport that are needed for mRNA vaccines. Compared to live attenuated vaccines, DNA vaccines have a safe clinical profile since the simplified DNA sequences lack the capacity to revert to a virulent form of the virus and there is no evidence of integration into host DNA as a safety concern. Furthermore, DNA vaccines have the flexibility to deliver more than one antigen, which are produced by the host in their native form to activate both humoral and cell-mediated immunity. In contrast to virally vectored vaccines, DNA vaccines do not elicit pre-existing immunity against the vector [1,2,5,6]. These advantages are especially desirable in mass vaccination campaigns during pandemics since vaccines can be produced quickly and distributed widely to areas where resources and logistical support are limited.

DNA administration began in the 1990s when Wolff et al. injected naked DNA plasmids into mice intramuscularly and detected local transgene expression up to 60 days after injection [7]. Wolff et al. and other groups found that rates of integration into host genomes are negligible and much lower than spontaneous mutation rates, with injected plasmid DNA present mostly in non-replicating, un-integrated circular form [1,7,8]. Additionally, DNA vaccination has not been found to induce enhanced production of auto-antibodies in pre-clinical and clinical trials, eliminating the concern regarding the development of auto-immune disease [9]. Routes of administration for DNA vaccines include intramuscular (IM), intradermal (ID), transmucosal, subcutaneous (SC), oral, intravenous, intranasal, and pulmonary [2,6].

Traditional DNA plasmids used for vaccination are of bacterial origin and contain the coding sequence(s) for immunogenic antigen(s) under the control of a strong eukaryotic promoter, a bacterial origin of replication, and a selectable marker [1,2,3,5]. Bacterial sequences, however, are not required for expression, could potentially cause a reduction in gene expression in mammalian cells, and also elicit safety concerns (such as the dissemination of antibiotic resistance genes into the host microbiota and induction of inflammatory responses that could enhance the immune response but may lead to silencing of expression). Recent DNA platforms have reduced amounts of bacterial sequences in attempts to increase antigen expression while alleviating safety concerns [1,2,5] and include minicircle DNA or linearized minimalistic, immunologically defined gene expression and doggybone DNA [1,5]. The mechanism of action of DNA vaccines involves a series of steps needed to induce an immune response. First, the DNA must enter the cell by crossing the outer cell membrane then translocate into the nucleus before being transcribed into mRNA. After transcription, the mRNA is exported from the nucleus to the cytoplasm for translation of antigens, which can then be expressed on the cellular surface. This process can happen in various cell types including antigen-presenting cells (APC), which leads to major histocompatibility (MHC)-I or MHC-II interactions and the cascade of events that elicit both humoral and cell-mediated immune responses following engagement with CD4+ and CD8+ T cells. In one pathway, transfected non-immune cells express antigens in the context of MHC-I molecules and engage CD8+ T cells; however, non-immune cells do not efficiently present antigens, and this pathway is not the main driver of immune activation [2]. A second pathway involves non-immune cells releasing antigenic peptides in exosomes or apoptotic bodies that are endocytosed by APC and then processed and cross-presented on MHC-II molecules for engagement and activation of CD4+ T cells that differentiate into T helper cells. These T helper cells then engage B cells, leading to the production of antibodies and differentiation into memory B cells. In the third pathway, transfected APC produce antigens that are processed and presented on both MHC-I and MHC-II molecules, activating both CD4+ and CD8+ T cells, leading to the production of antibodies and differentiation of CD8+ T cells to cytotoxic T lymphocytes (CTL) that can recognize and kill infected cells [1,2,3]. Activated T cells also secrete a variety of molecules during this process, including cytokines (IFN-γ, TNF-α, and IL-2), chemokines, cytotoxic granules, apoptosis-inducing molecules, and others. In addition to adaptive immune responses, DNA also induces innate immunity through host pattern recognition receptors such as Toll-like receptor 9 which recognizes un-methylated CpG motifs present in bacterial plasmid DNA, leading to the activation of innate pathways and production of type I IFN and other inflammatory cytokines [3]. Due to this activation, CpG motifs are sometimes added to DNA vaccines in an effort to boost immunogenicity.

DNA has been utilized in vaccines and therapeutics for infectious diseases, cancer, allergies, autoimmune diseases, and gene therapy [2,10]. Multiple vaccines have been licensed for veterinary applications, including against West Nile virus (WNV) in horses, malignant melanoma in dogs, infectious hematopoietic necrosis and pancreas disease in fish, avian influenza in chickens, and growth hormone-releasing hormone in pigs [2,3,11,12]. Prophylactic DNA vaccines that have been recently investigated in clinical trials include those against hantaviruses [13], alphaviruses [14], human immunodeficiency virus (HIV) [15], influenza [16], Zika virus (ZIKV) [17], WNV [18], Ebola virus [19,20], and severe acute respiratory syndrome coronavirus 2 (SARS-CoV-2) [21,22]. Non-viral indications for investigational prophylactic DNA vaccines in pre-clinical studies or clinical trials include *Staphylococcus aureus*, tuberculosis, *Cryptosporidium parvum*, malaria, and toxoplasmosis [23,24,25,26,27,28]. The first DNA vaccine for administration in humans was licensed in 2021, when Zydus Lifesciences obtained approval from the Drug Controller General of India for *Restricted Use in Emergency Situation* of its coronavirus disease 2019 (COVID-19) vaccine, ZyCoV-D using the PharmaJet Tropis intradermal needle-free injection system (NFIS) [29,30,31].

To elicit an effective immune response, DNA vaccines must overcome cellular and nuclear membrane barriers to enter the cell [1,2,5,6]. Additionally, nucleases rapidly degrade DNA once injected. Consequently, only a small amount of DNA injected via needle and syringe (NS) is taken up by cells while the majority remains in the extracellular space, leading to poor antigen expression and an ineffective immune response. To address the main challenge of low immunogenicity, considerable research has been devoted to developing techniques to boost the immune response following DNA vaccination. Strategies to increase immunogenicity include modifying the DNA construct (codon optimization, promoter selection, genetic adjuvants, addition of APC- or T cell-targeting sequences, removal of bacterial sequences), traditional aluminum adjuvants, physical delivery methods to deliver the DNA intracellularly (electroporation, particle-mediated epidermal delivery, NFIS, microneedles), chemical delivery methods or formulations (lipid or PLGA nanoparticles, PEI cationic polymers), and prime–boost vaccination schedules (either homologous or heterologous) [1,2,3,5,10].

This review focuses on broadly commercially available NFIS as a method to enhance immunogenicity and clinical benefits of prophylactic DNA vaccines. Currently, the PharmaJet Tropis and Stratis are the only NFIS commercially available in this specific market. Published scientific literature of prophylactic DNA vaccine studies from government, academic, and pharmaceutical groups featuring the use of these devices are reviewed here, with a focus on safety, immunogenicity, and clinical efficacy. The PubMed search engine was used to search for published articles, focusing on NFIS used for prophylactic DNA vaccine delivery. Due to these search conditions, the studies reviewed here focus on viral diseases and are further divided into sections comparing NFIS to electroporation and particle-mediated epidermal delivery, and NFIS delivery of pandemic and emerging and infectious viral diseases. Comparisons of NFIS to NS delivery are discussed when conducted within each of the studies reviewed.

## 2. Physical Delivery Methods to Improve Intracellular DNA Vaccine Entry

### 2.1. Particle-Mediated Epidermal Delivery

**Figure 1:**
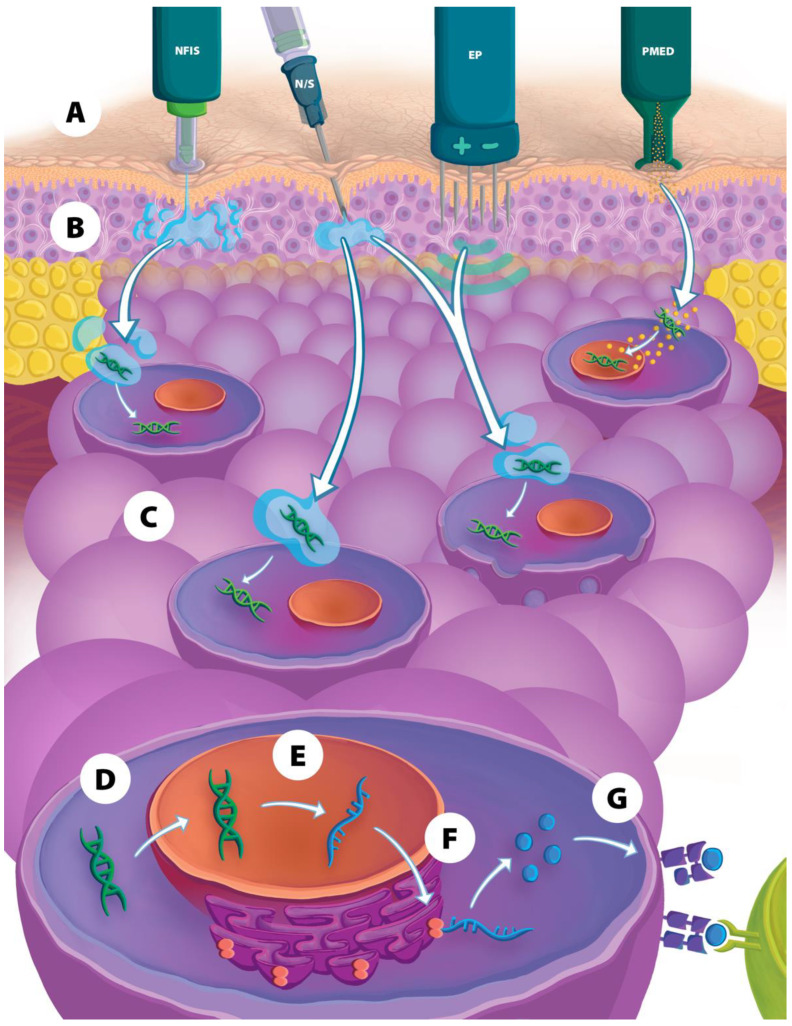
Overview of physical delivery methods used for DNA vaccines. (**A**) Depiction of four types of physical delivery methods. (**B**) During injection, the DNA vaccine is inserted into either the intradermal, subcutaneous, or intramuscular tissue layer, (**C**) then DNA is transported into the cell, (**D**) and into the nucleus, (**E**) where the DNA is transcribed to mRNA, (**F**) then translated in the cytoplasm into antigenic peptides that are processed and (**G**) presented on the cellular surface to various immune cells for the induction of a humoral and cellular immune response. NFIS = needle-free injection system; N/S = needle and syringe; EP = electroporation; PMED = particle-mediated epidermal delivery.

Particle-mediated epidermal delivery (PMED), also known as “gene gun” delivery, involves bombardment of DNA-covered gold particles through intradermal or mucosal routes with the use of ballistic devices (Figure 1) [6,9,10]. Since DNA is introduced intracellularly, less DNA is required to induce an immune response [6]. Studies have shown that PMED vaccine delivery induces a Th2-based response and poor cell-mediated immunity compared to other delivery methods [6,9,10,32,33]. Only a small amount of DNA can be carried by each gold bead, thus necessitating multiple vaccinations at different skin surfaces per timepoint for larger vaccination doses. Other disadvantages include difficulty in storing drug product, conducting potency testing, and instability of DNA on the beads [10,34]. Due to these limitations, PMED delivery is no longer used for human DNA vaccination [9], although the technology is being re-evaluated in pre-clinical studies.

### 2.2. Electroporation

Electroporation (EP) has been used to increase delivery of DNA vaccines intracellularly to enhance immune response (Figure 1) [3,6,9,10]. Following injection of the DNA vaccine with a NS, an electrical current is applied via one or more needles that have been inserted adjacent to the vaccination site. The electrical impulses increase cell membrane permeability via the generation of temporary pores, among other suggested pathways, that allow DNA to enter the cytoplasm and translocate into the nucleus, thereby increasing transfection rates 100- to 2000-fold [6,9,10,35,36]. The application of electrical pulses also causes local cell death and tissue damage, which leads to the release of damage-associated molecular patterns that have a natural adjuvant effect [6]. EP also leads to the production of inflammatory cytokines and local APC and T-cell migration [37,38]. In this manner, immune responses after EP can be increased 10- to 1000-fold [37,39] and, similar to PMED, less DNA is required to elicit an effective immune response [6]. Adverse events most commonly seen with EP are pain and bleeding at the injection site [9]. Disadvantages of EP include the need for a power supply to generate electricity, difficulties in IM injection in obese patients, costly and cumbersome equipment, the need to optimize electrical current regimens for different tissues, and potential deleterious effects resulting from tissue damage following EP [9,10]. Some examples of currently available EP devices include the CELLECTRA line (Inovio Pharmaceuticals, Plymouth Meeting, USA), TriGrid (Ichor Medical Systems, San Diego, USA), AgilePulse (BTX, Holliston, USA), CUY21EDIT (Nepa Gene, Ichikawa, Japan), and the CLINIPORATOR (IGEA, Carpi, Italy). Recently, EP has been used in clinical trials to deliver DNA vaccines for COVID-19 [21,22], HPV [40], MERS [41], and various cancers [42,43].

### 2.3. Needle-Free Injection Systems

Early-generation NFIS were developed in the 1930s and were used to combat infectious disease pandemics (e.g., smallpox, polio, and measles) and administer therapeutics and other products [35,44,45,46]. NFIS inject vaccines and other therapeutics in a fraction of a second via a high-velocity jet stream through a narrow orifice at high pressure, effectively piercing the skin and delivering injectate into either ID, IM, or SC tissue without the need for a needle (Figure 1) [35,44,47]. Types of NFIS have included gas- or spring-powered devices that do not require a battery source and deliver either a single injection at a time or multiple injections in a consecutive manner (multi-use injectors) [35,48]. These earlier multi-use injectors were found to cause cross-contamination and, by the 1990s, were largely supplanted by disposable syringe NFIS to mitigate this issue [35,44,48,49].

New-generation NFIS provide a vast improvement upon previous jet injectors. They are portable, easy to operate, and can deliver thousands of injections with single-use, disposable syringes that are also auto-disabling. An important advantage seen in studies using modern NFIS is that the immunogenicity elicited by jet delivery of vaccines may be enhanced compared to traditional vaccine delivery methods, partly due to the wider dispersion pattern of the injectate within tissues and more efficient delivery into cells in the case of DNA vaccines [35,47,50,51,52,53]. Furthermore, researchers have found dose-sparing effects from vaccines administered with NFIS, whereupon smaller doses administered with NFIS elicit similarly increased immunogenicity and protective efficacy compared to full doses delivered via either NFIS or NS [54,55,56]. Dose sparing of vaccines also represents the ability for “surge capacity” to rapidly respond to public health needs and emergencies without requiring the expansion of manufacturing capacity and scale. NFIS deliver consistent and accurate injection volumes at faster injection times [56,57,58,59,60] and significantly reduce vaccine wastage compared to NS [57,58,60]. As the name implies, another advantage of NFIS is the elimination of needle use and associated needle-phobia felt by patients (which can be up to 32% prevalent) [57,61,62], as well as the elimination of the risk of needle re-use and needle-stick injuries (which can cost up to $5000 per case in the USA and can be prevalent in over 56% of healthcare workers worldwide throughout their careers) [63,64], and the necessity of sharps disposal, which also incur significant costs [35]. Additionally, patients report a preference and may react less to NFIS delivery compared to traditional delivery methods [56,57,59,60]. Thus, NFIS are considered an attractive alternative to NS in mass vaccination campaigns [57,59,60,65], especially in more remote or rural areas with limited resources where delivery via NFIS can also be more cost-effective than NS delivery [66]. Indications for which NFIS have recently been used to deliver prophylactic DNA vaccines include COVID-19, HIV, Zika, HPV, influenza, dengue, polio, and measles–mumps–rubella viral infections [17,30,54,57,67,68,69,70,71,72,73], staphylococcal and tuberculosis bacterial infections [25,27], and malarial and cryptococcal parasitic infections [23,28].

Disadvantages of NFIS in general include a lack of familiarity among healthcare workers in settings where they are not routinely used, a potential increase in cost compared to NS (which is highly dependent on the specific market in terms of high-income countries compared to low- and middle-income countries, health application area, and demand volume or manufacturing scale), variability in injection penetration due to differing tissue properties, a potentially bulky device design, pre-set injection volume limits, reliance on an external power source, the inapplicability to intravenous administration, unavailability in a pre-filled format, a potential increase in injection site pain and reactogenicity based on route and type of injectable, and potentially lowered immunogenicity or clinical efficacy compared to other physical delivery methods which could be due to a complex variety of factors including vaccine dosage, route of administration, animal model selection, and others [35,44,45,46,47].

The PharmaJet Tropis (for ID administration) and Stratis (for IM/SC administration) are the only NFIS currently commercially available for immunization programs. Both have United States (US) Food and Drug Administration (FDA) 510(k) marketing clearance, Conformitè Europëenne (CE) Mark, and World Health Organization (WHO) PQS certification required for United Nations (UN) agency procurement. Tropis and Stratis use disposable, needle-free syringes that can be used to draw vaccines from standard vaccine vials using a disposable adapter. Stratis is licensed as a delivery method for the Afluria Quadrivalent seasonal influenza vaccine (Seqirus) in the US [74] and Tropis is licensed for the ZyCoV-D COVID-19 vaccine (Zydus Lifesciences) in India [31,75]. For additional discussion of jet injection and new-generation NFIS, see previous review articles [35,46].

## 3. Comparison of NFIS to EP and PMED Vaccine Delivery

Until recently, EP was the physical delivery method most studied to enhance the immunogenicity and efficacy of DNA vaccines. NFIS offer advantages when compared to EP, including not requiring an external power source and cumbersome equipment needed by EP to deliver electric pulses to facilitate DNA entry into cells, enhanced convenience in field situations, the elimination of the adverse experience that patients may feel associated with electrical pulses, and the elimination of needle use during injection procedures [76,77]. For reasons mentioned earlier in this review, PMED research has waned over time and is no longer routinely used. When comparing NFIS to these previously established methods, initial pre-clinical experiments typically measure immunogenicity and clinical response following vaccination and challenge, in addition to ease of use and patient tolerability, before advancing to clinical trials. As reviewed in this section, NFIS responses have tended to be non-inferior to EP and research staff report that NFIS is easier to use and more tolerable than EP and PMED. The pre-clinical studies reviewed in this section are summarized in Table 1 below. The clinical picture is less clear; however, a phase 1 trial (NCT01502345) evaluating Hantaan, Puumala, and Hantaan/Puumala DNA vaccines delivered by Ichor IM EP [78] resulted in neutralizing antibody responses comparable to those elicited in a similar phase 1 trial (NCT0277676) evaluating these hantavirus DNA vaccines delivered by the PharmaJet Stratis device (unpublished data). The Hantaan DNA vaccine plasmids in the two studies were slightly different in that the plasmid used in the PharmaJet study was codon-optimized; however, the Puumala DNA vaccine plasmids were identical and the dose administered was the same. Comparing day 84 (1 month after third vaccination) samples, the seroconversion (PRNT50 ≥ 40) rate and PRNT50 geometric mean titer (GMT) for the Puumala DNA vaccine delivered by IM EP vs. PharmaJet Stratis were 67%, GMT = 166 and 88%, GMT = 627, respectively.

The Institut National de la Recherche Agronomique evaluated a porcine reproductive and respiratory syndrome virus (PRRSV) DNA vaccine prime (delivered via EP or Tropis NFIS) in a heterologous prime–boost regimen with a modified live virus (MLV) vaccine boost (delivered by NS) in pigs and compared Tropis results with EP delivery (Table 1) [52]. Delivery via NFIS was more efficient at transfecting skin cells in vivo, was as potent in eliciting binding antibody responses, and resulted in decreased clinical changes after infection compared to EP. Preliminary experiments found significantly increased luciferase plasmid transfection efficiency in biopsied skin cells and a similar microanatomic pattern of expression in the ID Tropis group, with the EP group showing a significantly higher local inflammatory reaction. The heterologous DNA-MLV regimen showed increased effectiveness compared to homologous boosting in terms of specific binding antibody, neutralizing antibody, and IFN-γ T-cell responses. Similar binding antibody titers were elicited with the two delivery methods, with neutralizing antibody responses that were slightly higher in the EP group and higher T-cell responses in the NFIS group. Body temperature measurements rose the least with NFIS vaccination compared to EP, indicating better protection from clinical infection. The authors noted that combining NFIS delivery with immune-boosting formulations (such as cationic poly-lactoglycolide acid (PLGA) nanoparticles) or other types of adjuvants could further increase immunogenicity, in addition to different T-cell antigen selection [52].

Hooper et al., at the United States Army Medical Research Institute of Infectious Diseases (USAMRIID), have used NFIS to administer DNA vaccines against various viral infections. This group assessed hantavirus DNA vaccines in a Syrian hamster model and found enhanced immunogenicity, increased local antigen expression in tissues, and less injection site pathology in Tropis NFIS groups compared to PMED and NS delivery (Table 1) [79]. Although the Tropis system is normally used to deliver ID in larger animals and humans, the group developed a method to deliver IM and SC injections with the device in the smaller hamster model. Hamsters vaccinated with a Seoul orthohantavirus (SEOV) DNA vaccine showed that NS immunization was poorly immunogenic, while the opposite was found for Tropis injection, which showed induction of high neutralizing antibody titers after the second vaccination. While there was no significant difference in antibody titers between Tropis SC and IM injection, the IM route induced less variability in responses. Vaccinated hamsters were protected from SEOV infection after challenge. NS administration showed histopathological injection site changes whereas none were found in the NFIS group. NFIS injection also showed increased antigen expression in skin layers compared to NS, and only NFIS-group animals showed expression in connective tissue adjacent to inguinal lymph nodes. A comparison of the immunogenicity of SEOV and other DNA hantavirus vaccines used in this study to historical PMED vaccination data was conducted and found significantly increased immunogenicity and seroconversion rates for codon-optimized vaccines when delivered by NFIS and not by PMED. Additionally, the authors mentioned that animal model selection may affect immune response (in reference to using hamsters, rabbits, or NHP for their studies) [79].

Most recently, this USAMRIID group published findings of their “4pox” DNA vaccine against vaccinia virus in rabbits and found that NFIS yielded similar and increased immunogenicity compared to EP and NS, respectively (Table 1) [77]. Binding antibodies were elicited against all antigens, with no statistical difference in titers between NFIS and EP for three out of four antigens and higher with IM EP for the fourth antigen. Both Stratis and Tropis NFIS elicited antibody titers that were significantly higher than ID NS for three out of four antigens and higher with Tropis for the fourth antigen, showing the benefit of using Tropis ID over Stratis IM in this case. NFIS also produced high titers of functional antipox antibodies that were not significantly different than EP and higher than ID NS. The authors mentioned that while IM EP elicited the most robust immune responses, the increased convenience of NFIS, high titer development, and superiority to NS make NFIS ideal for field applications. Future studies from the group will focus on optimizing vaccination dosage and schedule with NFIS delivery prior to viral challenge [77].

A DNA influenza vaccine targeted to antigen-presenting cells (APC) was evaluated by Grodeland et al. at the University of Oslo and antibody responses elicited by ID delivery with either Tropis or EP were compared [76]. Pigs were immunized with various doses of DNA plasmid, with a single injection of 100 μg of vaccine eliciting significant and comparable specific antibodies by either delivery method. Upon boosting, however, animals produced higher (though not significant) titers of neutralizing antibodies with Tropis compared to EP. Studies comparing the effect of targeting to MHC class II were conducted with NFIS only and showed that less DNA was required to induce neutralizing antibody responses with the targeted vaccine compared to the un-targeted one. The authors also remarked that injection by NFIS was virtually “pain-free” compared to EP and that jet delivery could substitute for EP administration [76]. The group conducted a follow-up study in NHP using Tropis only, reviewed in Section 4.2 below [81].

In another study comparing NFIS to EP, Williams et al. used a tetravalent dengue virus (TVDV) DNA vaccine in NHP and compared immunogenicity and protective efficacy between routes, doses, and delivery methods [80]. The authors found that EP and ID delivery had the best responses, with ID delivery showing dose-sparing effects compared to IM for neutralizing antibody titers and concluded that either EP or Tropis delivery should be further explored in humans. All vaccinated groups showed full neutralizing antibody seroconversion. These antibody responses were durable and were maintained for more than a year in all of the animals in the high-dose ID groups regardless of delivery method. EP and ID delivery yielded the highest neutralizing antibody titers, with no statistical difference between EP and high-dose Tropis groups. All of the groups developed IFN-γ T-cell responses, with the high-dose IM EP group showing 100% positivity. Peak IFN-γ responses trended higher for the EP groups but were not significantly different than the high-dose Tropis group. More animals in the EP groups showed a memory B-cell response and percentages of DENV-specific memory B cells were highest in the ID groups, with no statistical differences found between the groups. Following viral challenge, animals in EP and Tropis groups had significantly lower peak viremia than controls. Animals in the ID EP groups had the lowest number of days showing viremia, followed by the high-dose IM EP and Tropis groups. The authors noted that a limitation of NFIS use in this study was the limited injection volume that could be administered with one injection with the Tropis device [80]; however, this limitation is commonly overcome by administering more than one injection per timepoint which could be relatively easy to do as patients have reported a preference of NFIS over NS and EP delivery [56,57,59,60].

## 4. NFIS Delivery of DNA Vaccines for Pandemic Viruses

Pandemics provide an example of when vaccines need to be urgently produced and delivered at large scale to reduce viral transmission and incidence of disease. In this section, we review NFIS delivery of DNA vaccines for pandemic viruses, including COVID-19, influenza, and HIV. The studies are summarized in Table 2 below.

### 4.1. COVID-19

Research groups have tested multiple types of vaccines against SARS-CoV-2, the causative agent of COVID-19, with mRNA, adenoviral-vectored, and protein-based platforms having obtained Emergency Use Authorization (EUA) in the US [91]. Joining this effort to produce prophylactic vaccines against COVID-19, Zydus Lifesciences obtained approval for *Restricted Use in Emergency Situation* of COVID-19 from the Drug Controller General of India for ZyCoV-D, the world’s first approved human DNA vaccine, to be administered exclusively with the Tropis NFIS [29,30,31]. As the studies in this section show, NFIS delivery of COVID-19 vaccines induces increased immunogenic effectiveness and protective efficacy compared to NS in both pre-clinical and clinical studies and is amenable for novel DNA platforms (Table 2).

For the ZyCoV-D vaccine, pre-clinical studies began in mice and guinea pigs using NS before progressing to rabbits and NHP with the addition of Tropis delivery. Dey et al. vaccinated rabbits (Table 2) and showed production of significant binding and neutralizing antibody responses, with the authors remarking on the usefulness and efficiency of the Tropis NFIS [82]. Subsequently, Yadav et al. tested ZyCoV-D in NHP and compared immunogenicity and protective efficacy of Tropis to NS delivery [83]. The high-dose Tropis group was the most effective and efficacious, with lower doses and NS delivery considered ineffective. Animals immunized with Tropis had persistent and significantly increased binding and neutralizing antibody responses that developed earlier than the NS group, with none of the low-dose NS-vaccinated animals showing a response. Following challenge, development of binding and neutralizing antibodies followed the same trends. B-cell population numbers also trended higher in the high-dose Tropis group and there was a significant increase in the percentages of lymphocytes and IL-8 in this group, indicating possible vaccine-induced lymphoid proliferation and immune response [83]. In terms of protective efficacy, animals in the high-dose Tropis group showed the highest reduction in viral loads and earliest viral clearance. The authors noted a limitation in their study, where they collected samples from control animals until 7 days post-challenge as opposed to vaccinated animals, which were sampled up to 15 days post-challenge [83].

In the ZyCoV-D phase 1/2 trial in India, groups of healthy adults were vaccinated with the same doses and schedule as the NHP studies and immunogenicity between Tropis and NS delivery was compared (Table 2) [29]. Phase 1 results showed that ZyCoV-D was safe and well-tolerated, with similar occurrence of solicited adverse events (AE) reported for all groups that were mild to moderate in intensity with no abnormal clinical parameters observed. Binding and neutralizing antibody end-point seroconversion rates showed a dose-dependent increase in titer with Tropis eliciting higher responses compared to NS. IFN-ɣ T-cell responses trended higher and were sustained until the end of the study for the Tropis groups as opposed to the low-dose NS group, although the authors noted that they were limited by sample size in the different groups and were unable to make definitive conclusions regarding the cellular immune response in the participants. The authors found that phase 1 trial results correlated well with the previous NHP studies. Limitations mentioned about the study were the inclusion of only male participants and that participants were followed only until 84 days post-vaccination [29]. Khobragade et al. published interim phase 3 clinical trial results using only the Tropis NFIS to compare safety, effectiveness, and protective efficacy of ZyCoV-D to placebo in a wider selection of participant age groups and those with comorbidities (Table 2) [30]. The vaccine was safe and well-tolerated with no difference between groups in AE. ZyCoV-D groups had higher binding and neutralizing antibody seroconversion rates (significantly for neutralizing), concentration, and increase in titer compared to placebo. Cellular responses in the ZyCoV-D group were significantly increased compared to placebo and were over 13 times higher than baseline after two vaccinations and still increased by nearly 10-fold later in the study. ZyCoV-D was found to be 100% efficacious in preventing moderate to severe disease and 64.9% against mild disease. The vaccine was considered to be efficacious against the Delta variant of concern (VOC) since it was circulating in India during the study. The authors added that the use of NFIS should decrease the occurrence of side-effects associated with NS administration, such as pain at the injection site. Limitations of this study mentioned by the authors included a predominance of male participants, a lack of efficacy analysis for certain subgroups, a lack of common hematological and organ function analyses, a small sample size, and a relatively short duration of study [30].

USAMRIID tested both doggybone and plasmid DNA vaccines in hamsters delivered IM via the Tropis NFIS only (Table 2) [84,85]. Initial experiments with their plasmid DNA vaccine, nCoV-S(JET), showed that it was immunogenic and protective against infection when delivered by the Tropis NFIS [85]. The group then used the doggybone DNA (dbDNA™) platform developed by Touchlight Genetics and designed the dbDNAS(JET) and stabilized dbDNAS(ST-JET) DNA vaccines based on the nCoV-S(JET) vaccine, administered to hamsters at two dose levels. They found that the dbDNA platform performed similarly to the plasmid construct when delivered by NFIS, with all three vaccines inducing cross-protective neutralizing antibodies. All but one of the vaccinated wild-type hamsters in the low-dose group produced neutralizing antibodies, with the nCoV-S(JET) and dbDNAS(ST-JET) vaccines producing the highest titers at either dose level, regardless of number of vaccines given. The immunosuppressed hamster model showed significant differences in body weight for all three vaccines at the higher dose in addition to reduced viral RNA, infectious virus, pathology, and antigen staining in the lungs, with dbDNAS(ST-JET) and nCoV-S(JET) performing better than dbDNAS(JET). The three vaccines induced cross-reactive neutralizing antibodies against D164G and other VOC, with dbDNAS(JET) showing the lowest titers. The authors commented that the results in this study likely represent disease in immunocompromised people and that future studies should be conducted in multiple animal models. Overall, these studies showed the feasibility of using the NFIS platform to deliver doggybone DNA vaccines and the authors remarked on the simplicity of using jet injection with the added benefits of increasing immunogenicity and dose sparing seen with this delivery method [84].

The pNTC-Spike plasmid DNA vaccine developed by Statens Serum Institut was tested in rabbits and NHP using both Tropis and Stratis and results were compared to NS administration (Table 2) [86]. In rabbits, ID and IM NFIS injection was well-tolerated and induced higher and more consistent responses than NS. Binding and neutralizing antibodies were detected after the first and second immunizations, respectively, and were increased with subsequent boosting. Antibodies generated by all delivery methods cross-reacted with multiple VOC, with NFIS groups eliciting neutralizing responses in up to 100% of vaccinated animals. T-cell cytokine responses were comparable between NS and NFIS groups. Follow-up studies in NHP were performed with the Tropis ID only and, similar to the rabbits, vaccination in NHP was well-tolerated and induced increasing binding and neutralizing antibody titers that cross-reacted with VOC. The NHP model showed increased potency compared to the rabbits. After viral challenge, vaccinated NHP showed a 2 log_10_ reduction in bronchoalveolar lavage (BAL) viral subgenomic mRNA and viral clearance in nearly all animals by four days post-challenge. Specific binding antibody titers were boosted and significantly increased after challenge compared to pre-challenge titers. The authors remarked that the increased potency of the vaccine in NHP was likely in part due to NFIS injection and that future strategies to increase immune response and protective effects of the vaccine could include a longer interval between boosting, using a heterologous boosting regimen, and increasing the dose amount in order to decrease the number of vaccinations required to achieve sufficient immunity [86].

SaudiVax vaccinated mice with their VIU-1005 DNA vaccine via a modified Tropis device, finding enhanced immunogenicity with an increased and long-lasting Th1-skewed humoral response and dose sparing with NFIS compared to NS (Table 2) [55]. Two to three vaccinations of the lowest dose via IM Tropis elicited significant binding and neutralizing antibody responses. Notably, two vaccinations with Tropis induced equivalent or higher antibody titers compared to three vaccinations with a higher dose via IM NS. Tropis vaccination induced seroconversion in all animals and also showed less variability in responses as opposed to NS. Tropis vaccination also induced elevated specific IgG2a and IgG2b, suggesting a Th1-biased response [55]. Memory T cells showed significant expression of IFN-ɣ, TNF-α, and IL-2 from CD8+ cells and high TNF-α levels from CD4+ T cells, which were significantly higher in the IM Tropis group compared to NS. Results for ID Tropis administration followed similar trends as the IM group. NS ID and SC injection failed to induce high levels of binding antibodies. The authors commented that NFIS delivery enhanced the immune responses in this study and that combining NFIS with EP delivery should also be investigated to determine if the combination of delivery methods would further enhance the immune response [55]. The group also tested their pVAX-S1 DNA vaccine in mice given IM with the modified Tropis (Table 2), showing similar results [87].

### 4.2. Influenza

Recent influenza DNA vaccines administered with NFIS (Table 2) have typically used hemagglutinin (HA) as the immunogen. Successful techniques used by these groups include heterologous boosting regimens and APC-targeting sequences. Cross-protection against heterologous influenza strains has also been shown with NFIS in efforts to induce broadly neutralizing antibodies against multiple strains.

The National Institutes of Health’s (NIH) Vaccine Research Center (VRC) tested a heterologous prime–boost regimen against influenza using plasmid DNA and ferritin nanoparticle vaccines in a phase 1 trial, showing that the heterologous regimen was well-tolerated and more immunogenic than homologous boosting (Table 2) [16]. Participants were given either a single vaccination with the H2HA ferritin nanoparticle vaccine via NS, a homologous prime–boost of the nanoparticle vaccine, or a heterologous prime–boost with an H2 DNA vaccine prime (via IM Stratis) and nanoparticle vaccine boost (via NS). Reactogenicity was generally mild to moderate in severity. Binding antibodies against H2 were detected in all groups, with H2-naïve adults showing higher-fold increases in group 1 HA stem-targeting antibodies in the heterologous regimen compared to homologous boosting. Anti-H2 neutralizing antibody levels also showed a higher fold-change after boosting in the heterologous regimen. The increase in neutralizing activity was durable up to 6 months after the boost and showed cross-reactivity against H5N1 in H2- naïve participants, again showing higher fold-changes in the heterologous regimen [16].

Following the success of their previous studies in pigs immunized against influenza using the Tropis device in comparison to EP (see Section 3 above) [76], Mooij et al. at the University of Oslo published findings on an APC-targeted plasmid DNA vaccine against H1N1 influenza in NHP using the Tropis NFIS only [81]. Overall, the authors showed that the vaccine was safe, induced both humoral and cellular immunity, and was protective in the NHP model. None of the vaccinated animals showed AE after vaccination, 100% seroconverted after the third vaccination, and the group showed significantly increased neutralizing antibody responses compared to the control group. IFN-ɣ T-cell responses were induced after one vaccination and continued to increase in all animals after boosting. After challenge with H1N1, viral loads were significantly reduced in the vaccinated group, with two out of six animals remaining negative throughout the study period and two others showing positivity at only one timepoint. Body temperature was also decreased in immunized animals and only showed one fever peak compared to controls. Anti-HA antibodies and CD4+ T-cell responses were significantly increased, with the two NHP that were completely protected from viremia showing the highest responses. The majority of cytokine-secreting cells detected were polyfunctional and secreted IFN-ɣ, IL-2, and TNF-α. Additionally, vaccinated animals showed reduced peak levels of IL-6, MCP-1, and IFN-ɣ compared to controls after viral challenge. The authors remarked that jet delivery with NFIS is “pain-free” and could be an effective tool to protect against seasonal and pandemic influenza viruses [81].

### 4.3. HIV

Dr. Williamson’s group at the University of Cape Town has developed DNA vaccines expressing HIV Gag virus-like particles and envelope (Env), which have been tested in rabbits using heterologous prime–boost regimens (Table 2) [89]. Rabbits were inoculated with two DNA primes followed by two to four poxvirus-vectored boosts and, in some studies, two HIV envelope protein boosts. Initially, the vaccines were administered IM using a NS [67,92,93]. In these experiments, none of the rabbits developed binding antibody responses to HIV Env after the two DNA vaccinations.

More recently, the immune response elicited following heterologous IM delivery of DNA and modified vaccinia Ankara (MVA) vaccines expressing HIV Gag and Env using Stratis was compared to NS (Chapman et al.; manuscript in preparation). A clear difference was seen in the immune response when the DNA vaccines were delivered by NFIS, with 6 out of 10 rabbits developing binding antibodies to HIV Env after Stratis vaccination compared to none in the NS group. In addition, 2/10 rabbits inoculated via NFIS developed Tier 1A neutralizing antibodies compared to none in the NS group. Vaccination with Stratis also appeared to improve responses to MVA vaccination, as three out of five rabbits in the Stratis group developed Tier 1B neutralizing antibody responses after the first MVA vaccination whereas only one rabbit did in the NS group, and an increase in the mean titers of Tier 2 and Tier 1B neutralizing antibodies was seen when the DNA and MVA vaccines were administered by NFIS. The effect of adjuvanting the DNA vaccines with CpG oligodeoxynucleotide (ODN) 1826 combined with Stratis delivery was found to further improve the immune response, with 10 out 15 rabbits developing binding antibody responses to HIV Env after a single DNA vaccination and all of them after two DNA vaccinations. Similar results were seen in other studies by this group when DNA vaccines were administered IM using Stratis both with and without CpG [88,89]. Overall, these studies showed that the immunogenicity of DNA vaccines delivering HIV antigens was significantly improved when delivered using NFIS as compared to NS.

In a study comparing poxviral-vector boosts, rabbits received a DNA prime followed by boosts with either MVA or lumpy skin disease virus (LSDV) in different sequences, given with Stratis only [89]. Serum binding and Tier 1A neutralizing antibodies were produced at similar levels in all of the vector combinations tried throughout the study period. Half of the animals developed binding antibodies against HIV Env after the two initial DNA vaccinations, which had not happened at this timepoint in previous studies with NS delivery. All of the rabbits also developed high levels of binding antibodies after the initial poxviral boost with either MVA or LSDV. MVA boosts induced significantly increased levels of Tier 1A neutralizing antibody compared to LSDV boosts. Additionally, the authors found increased Tier 1B and the presence of Tier 2 neutralizing antibodies depending on the order of alternating MVA and LSDV boosts. Overall, this study showed the immunogenicity of the LSDV vector boost and feasibility in using this type of vector for HIV vaccine development using NFIS. The authors noted that altering envelope sequences or increasing the time period between homologous boosting could increase immune responses [89].

The group also tested a bivalent plasmid DNA vaccine as part of a heterologous prime–boost regimen with a recombinant MVA-vectored vaccine (both given IM with Stratis) and an HIV Env protein vaccine produced either in mammalian or plant cells, given via NS [88]. Both groups showed comparable binding antibodies at the end-point. Staff administering these vaccines reported that the Stratis system was easier to use than NS and seemed to cause less discomfort in the rabbits (personal communication with Dr. Chapman). This study showed the feasibility of using plant-derived proteins as part of heterologous vaccination programs incorporating NFIS delivery.

Simpson et al., at the NIH National Institute of Allergy and Infectious Diseases, assessed the antigen-specific CD8+ T-cell repertoire (TCR) in NHP vaccinated with their plasmid DNA SIV-gag vaccine IM using the Stratis NFIS, finding that clonotypic hierarchies observed in vaccinated animals were similar to those in animals that were simian immunodeficiency virus (SIV)-infected [90]. After a limited period of antigenic exposure via vaccination, NHP showed public, shared, and unique TCRs that were tissue-specific and the clonotypic hierarchies observed were the same as those seen when NHP were SIV-infected. The data in this study help explain CD8+ T-cell residency phenomena following antigen exposure after vaccination with the Stratis NFIS [90].

## 5. NFIS Delivery of DNA Vaccines for Emerging Infectious Diseases

Research groups at USAMRIID and the NIH VRC have developed DNA-based vaccines for multiple emerging and re-emerging infectious diseases and viral agents that have potential use as bioweapons. Similar to results in previous sections, NFIS use has improved vaccine performance when compared to traditional NS delivery, in both pre-clinical and clinical trial settings. The studies reviewed in this section are summarized in Table 3 below.

### 5.1. Venezuelan Equine Encephalitis Virus (VEEV)

Dr. Hooper’s group at USAMRIID tested their pWRG/VEE plasmid DNA vaccine in NHP delivered via Tropis ID or Stratis IM and compared immunogenicity and protection from viral challenge (Table 3) [94]. Vaccinated animals showed significant protection from infection and although IM delivery induced more potent immune responses, animals in the ID group showed better protection from challenge. After vaccination, all animals produced binding IgG antibodies that were significantly higher in the IM group and a subset of NHP also produced IgA. Neutralizing antibody titers trended higher with IM delivery, although this was not statistically significant. IM delivery induced potent T-cell responses while ID delivery did not. After challenge, nearly all NHP remained aviremic with the exception of one animal in the IM group, and more animals in the IM group developed a fever. Brain histopathological lesions were correlated with body temperature data, with IM animals that had a sustained fever showing a high degree of encephalitis. The authors commented that ID delivery could induce higher antibody avidity and mucosal immunity, which could be the reason for the enhanced protective effect seen in the group. Future studies proposed by the authors include investigating the route of administration in the NHP model, increasing the ID group vaccine dose, further evaluating the T-cell responses in relation to protection from viral challenge, and assessing local mucosal immune responses [94]. A confirmatory study in NHP is in progress and a phase 1 clinical study is in preparation to evaluate the safety and immunogenicity of the VEEV DNA vaccine in humans at different doses and routes with NFIS.

### 5.2. Hantaviruses

Two hantaviral plasmid DNA vaccines were developed at USAMRIID and tested in rabbits and NHP, inducing increased immunogenicity when delivered with NFIS compared to NS (Table 3) [95]. The group tested Sin Nombre virus (SNV) and Andes orthohantavirus (ANDV) vaccines alone or in combination and delivered by four different methods: PharmaJet first-generation ID or IM devices (IDv1 and IMv1, respectively), IM Stratis, or IM NS. Rabbits immunized with the SNV vaccine (IDv1 or IMv1) showed production of neutralizing antibodies after the first vaccination, with IM titers increasing after boosting. The anti-SNV sera were protective in hamsters that were passively immunized and challenged with SNV. NHP immunized with the SNV DNA vaccine (IDv1 or IMv1) showed that the IM route was more immunogenic than ID. Similar neutralizing antibody responses were seen when SNV and ANDV vaccines were given either separately or combined in one syringe with the Stratis NFIS, indicating the feasibility of combined injection. Finally, the group tested the combined SNV/ANDV vaccine in NHP using IMv1, Stratis, or IM NS delivery. Both IMv1 and Stratis elicited significantly higher neutralizing antibody titers and in more animals than NS delivery. The authors remarked that NFIS are the least cumbersome and most pragmatic compared to other delivery methods used for DNA vaccination including EP and PMED and also noted animal model differences in how efficiently vaccines were delivered into the ID compartment [95].

The group then tested their hantaviral DNA vaccines formulated in lipid nanoparticles (LNP) in rabbits and NHP with the Stratis NFIS (Table 3) [96]. This study showed the feasibility of using up to 20-fold less DNA when LNP-formulated to induce increased immunogenicity and cellular uptake compared with un-formulated vaccines. Initial tolerability experiments in rabbits with the ANDV LNP vaccine showed a dose-dependent neutralizing antibody response that increased with subsequent boosts in all the doses tested except for the lowest. A head-to-head comparison of LNP- and un-formulated vaccines showed neutralizing antibody responses earlier that were increased 30-fold and less variable in the LNP group. Comparisons of ZIKV LNP- and un-formulated vaccines in NHP showed neutralizing activity in the LNP group while the animals in the un-formulated group did not. The group also performed in vitro experiments using LNP-formulated DNA that showed more efficient cellular expression of protein and monoclonal antibody production in the LNP groups, suggesting that increased cellular update of LNP could be inducing increased immunogenicity after NFIS delivery in the animal models. Further studies suggested by the group for DNA-LNP vaccines include testing multiple doses and vaccination schedules, evaluating vaccine protective efficacy after viral challenge, and measuring the durability of immune responses [96].

### 5.3. Zika

The NIH VRC developed two plasmid DNA vaccine candidates against ZIKV (VRC5283 and VRC5283) and delivered them IM via Stratis only in NHP then followed up with clinical trials after showing effectiveness in pre-clinical studies [17,97,98,99]. Initial experiments by Dowd et al. showed immunogenicity and protection from viral challenge in vaccinated NHP, with a correlation between neutralizing antibody response and protective effect (Table 3) [97]. A single dose of either vaccine elicited detectable binding and neutralizing antibodies. NHP that received two immunizations of either dose of either vaccine produced similar neutralizing antibody titers that were significantly higher than when NHP were vaccinated once. After challenge, nearly all NPH that received two vaccinations of either vaccine were protected from infection, compared to no NPH being protected that received one vaccination of VRC5288. Further studies suggested by the group included testing a different antigen design, different delivery methods, and heterologous vaccination regimens [97]. Afterwards, the group vaccinated NHP with VRC5283 via Stratis in a ZIKV-exposed pregnant macaque model and found that the vaccine protected animals against prolonged viremia and also improved fetal outcomes (Table 3) [98]. NHP non-pregnant females were immunized IM with Stratis, mated to achieve pregnancy, and then inoculated with ZIKV to develop fetal congenital Zika syndrome. NHP showed peak neutralizing antibody 2–4 weeks after the second immunization, with animals that did not become pregnant showing a durable neutralizing antibody response. After viral challenge, 5 out of 13 vaccinated pregnant females were aviremic, and those that showed viremia had a transient and significantly reduced viral load (100-fold) that was undetectable by 14 days post-challenge. NHP that did not show viremia had higher levels of neutralizing antibodies. These results suggested that DNA vaccination with VRC5283 prior to pregnancy either reduced or prevented Zika viremia in pregnant macaques [98]. When challenged during gestation, vaccinated dams quickly mounted anamnestic antibody responses, remained aviremic, and showed similar binding and neutralizing antibody titers in fetal cord blood (indicating transplacental antibody transfer). Additionally, vaccinated females showed significantly increased and specific CD4+ T-cell activity. None of the vaccinated dams had fetal loss or showed viral RNA in amniotic fluid or in fetal tissues. Less fetuses from vaccinated females showed histopathological changes compared to the control group. Limitations for this study included a small sample size, study design (timing of mating and viral challenge, frequency of procedures on the pregnant animals and fetuses), length of measuring immune responses, and lack of monitoring newborn animals into adulthood [98].

Both VRC5283 and VRC5288 were advanced to a phase 1 clinical trial that showed the safety and enhanced immunogenicity of VRC5283 when delivered IM with Stratis compared to NS (Table 3) [17]. Participants were vaccinated with either one full dose (VRC5283 or VRC5288, both via NS) or split doses (VRC5283 with NS or Stratis). Both vaccines were safe and well-tolerated, with only mild to moderate reactogenicity. Stratis delivery induced the highest neutralizing antibody responses and achieved 100% seropositivity, while NS delivery reached 77–93% seropositivity. The authors also found that split-dose vaccination induced higher neutralizing activity. CD4+ and CD8+ T-cell total cytokine responses were also the highest in the Stratis NFIS group. The authors noted the limitation of a small sample size typical of phase 1 clinical trials. Based on these results, the group advanced the VRC5283 candidate to a phase 2 efficacy trial with Stratis as the only delivery method [17].

## 6. Conclusions and Future Prospects

New-generation NFIS offer several important benefits compared to traditional NS delivery of DNA-based vaccines. Increased immunogenicity is sought after by prophylactic vaccine developers and has been shown in the studies reviewed here [17,29,55,77,79,83,95]. NFIS typically yield comparable immunogenicity to EP [52,76,77] and increased responses compared to NS, which can sometimes fail to induce effective immunogenicity [29,55,83]. This is not surprising, considering that NFIS can more efficiently deliver DNA into cells for production of desired antigens. The increased immunogenicity of NFIS can be partially explained by increased gene expression of injected DNA [52,79]. This increased immunogenicity is expected to translate into enhanced protective efficacy, as was seen in the ZIKV vaccine studies conducted by the NIH VRC [97,98]. When comparing EP and NFIS, there are added benefits of NFIS, including increased practicality, ease of use in field situations, and a more comfortable patient experience. NFIS use has also shown less intra-group variability in immune responses [55,79]. Both humoral and cellular immunity can be effectively induced with NFIS delivery [17,29,30,52,55,81,83,87,94,98], in addition to producing unique types of immune responses such as a Th1-biased response with COVID-19 vaccines [55,86,87]. Additionally, studies discussed here show that pre-clinical results can translate to clinical trial results, with ZyCoV-D as an example, where results observed in NHP were repeated in clinical trials [29,83]. Pre-clinical animal models also show that vaccines delivered with NFIS can be as effective as other delivery methods in special clinical states, such as during immunosuppression and pregnancy [84,85,98].

In relation to DNA manufacturing, the studies reviewed here show that NFIS have dose-sparing effects that decrease the amount of DNA needed to induce an effective immune response, which can be critical during pandemic responses where DNA vaccines need to be produced rapidly at a large scale [55,94]. ID delivery in particular can yield this effect, as skin has abundant immune cell populations that could yield increased immunogenicity and protective effects with less vaccine than when delivered IM [77,80,94]. This was the case with ZyCoV-D, which was produced in an expedited manner and for which NFIS devices and consumables production was scaled up quickly to meet demand at mass vaccination quantities [29,30]. Indeed, NFIS have been used in mass vaccination campaigns against other viral infections and have been shown to be more cost-effective than NS administration [57,59,66]. The ability to combine vaccines into one NFIS syringe and induce a similar immune response as when vaccines are administered separately bring additional cost savings and convenience [95].

Other techniques have been combined with NFIS to boost vaccine immunogenicity and protection. Modern technologies such as the doggybone DNA platform and LNP formulation of DNA have shown success with NFIS [84,96]. Compared to conventional plasmid DNA vaccines, synthetic linear doggybone DNA can be produced more rapidly at GMP grade, enhances safety by eliminating bacterial genes found in plasmid DNA, and yields a smaller product compared to plasmid DNA. The ability of NFIS to deliver dbDNA vaccines and induce an effective immunogenic and protective effect positions this technology as an attractive option in designing vaccination regimens, especially in pandemic situations [84]. NFIS delivery of LNP-formulated DNA vaccines can lead to a safe and much more potent immune response at a faster rate and with less DNA than un-formulated vaccines, as reviewed here [96]. Although not covered in this review, a rabies mRNA vaccine that was complexed with the cationic protamine protein was delivered with both Tropis and Stratis in a phase 1 clinical trial, successfully inducing an effective immune response, while NS did not [54]. Heterologous prime–boost regimens employing a DNA vaccine prime with viral vectors or ferritin nanoparticle vaccine boosts have shown enhanced responses compared to homologous boosting [16,52]. Using molecular sequences to target antigens to APC has also shown success with NFIS delivery [52,76,81]. Co-administering DNA vaccines with adjuvants such as CpG ODN is also feasible with NFIS and boosts the immune response [88].

NFIS vaccine delivery, however, is not always successful and the devices can carry certain limitations depending on their design (reviewed in Section 2.3). Reasons for poor immunogenicity or clinical efficacy compared to other delivery methods could be complex and include an interplay between the animal species selected, the sequence of the DNA construct and codon optimization, use of molecular or traditional adjuvants, injection route (ID, IM, SC), vaccine dosage, vaccination schedule, use of heterologous boosting, whether immune-boosting formulations such as LNP were used, and other factors.

In summary, NFIS are easy and safe to use, with proven tolerability through clinical development programs and mass immunization campaigns while offering the potential to enhance the performance of classical and modern vaccine platforms. Therefore, these devices should be considered when designing DNA and other nucleic acid-based vaccination programs. Currently, variable-volume NFIS are under development at PharmaJet which would allow greater flexibility in delivering prophylactic vaccines. As mentioned in Section 1, DNA vaccine delivery with NFIS has been investigated for other indications (such as cancer and Alzheimer’s disease) not covered in this review. A publication reviewing therapeutic DNA vaccine delivery with commercially available NFIS is planned for future publication.

## Figures and Tables

**Table 1:** Studies comparing plasmid DNA vaccine delivery with NFIS, EP, or PMED.

Indication	Name (Immunogen)	Species	Device (Route)	EP Device or PMED	Dose	Vaccine Schedule	Vs. NS	NFIS Notes
PRRSV [52]	N, Nsp1β, RdRp	Pig	Tropis (ID)	CUY21EDIT	0.4 mg *	0, 34 d C: 63 d	No	APC-targeted vaccine Heterologous prime–boost with MLV vaccine NFIS increased gene expression
Hantavirus (various) [79]	M	Hamster	Tropis (SC, IM)	PMED	0.2 mg, 0.42 mg	0, 4, 8 wk C: 21 wk	Yes	IM NFIS showed less variability NFIS less pathology than NS NFIS more staining, expanded expression than NS NFIS more immunogenic than PMED and NS
Poxvirus [77]	4pox (L1, A27, B5, A33)	Rabbit	Tropis (ID) Stratis (IM)	TriGrid	0.4 mg	0, 4, 8 wk	Yes	NFIS non-inferior immunogenicity to EP, more immunogenic than NS NFIS suitable for field use Tropis had broader immunogenicity than Stratis
Influenza [76]	HA	Pig	Tropis (ID)	AgilePulse	0.025 −0.4 mg	0, 3–4 wk	No	APC-targeted vaccine NFIS comparable immunogenicity to EP NFIS injection “pain-free”
Dengue [80]	TVDV (Pre-M, E)	NHP	Tropis (ID) Stratis (IM)	TriGrid	1 mg, 5 mg	0, 28, 91 d C: 392 d	No	EP, ID delivery had highest nAb Dose-sparing with ID delivery

* = total dose administered in multiple injections at the same timepoint; NFIS = needle-free injection systems; EP = electroporation; PMED = particle-mediated epidermal delivery; vs. = versus; NS = needle and syringe; PRRSV = porcine reproductive and respiratory syndrome virus; N = nucleocapsid; Nsp = non-structural protein; RdRP = RNA-dependent RNA polymerase; ID = intradermal; d = day; C = challenge; APC = antigen-presenting cell; MLV = modified live virus; M = medium gene segment; SC = subcutaneous; IM = intramuscular; wk = week; HA = hemagglutinin; TVDV = tetravalent dengue vaccine; Pre-M = pre-membrane; E = envelope; NHP = non-human primate; nAb = neutralizing antibodies.

**Table 2:** Pandemic virus plasmid and doggybone DNA vaccines administered with NFIS.

Indication	Name (Immunogen)	Species	Device (Route)	Dose	Vaccination Schedule	Vs. NS	NFIS Notes
COVID-19 [82]	ZyCoV-D (Spike)	Rabbit	Tropis (ID)	0.5 mg	0, 2, 4 wk	No	NFIS useful and efficient
COVID-19 [83]	ZyCoV-D (Spike)	NHP	Tropis (ID)	1 mg, 2 mg *	0, 4, 8 wk C: 15 wk	Yes	Dose-dependent responses NS ineffective
COVID-19 [29]	ZyCoV-D (Spike)	Human Ph 1/2	Tropis (ID)	1 mg, 2 mg *	0, 4, 8 wk	Yes	Similar results as NHP study [83]
COVID-19 [30]	ZyCoV-D (Spike)	Human Ph 3	Tropis (ID)	2 mg *	0, 4, 8 wk	No	Significant immunogenicity 100% protection against moderate/severe disease, Delta VOC NFIS can decrease injection side-effects
COVID-19 [84]	nCoV-S(JET), dbDNAS(JET), dbDNAS(ST-JET) (Spike)	Hamster	Tropis (IM)	0.05 mg, 0.2 mg	0, 3 wk C: 6 wk	No	Synthetic doggybone DNA platform immunogenic and protective Immunocompromised model
COVID-19 [85]	nCoV-S(JET) (Spike)	Hamster	Tropis (IM)	0.2 mg	0, 3 wk C: 6 wk	No	Immunocompromised model NFIS simple to use
COVID-19 [86]	pNTC-Spike (Spike)	Rabbit NHP	Tropis (ID) Stratis (IM)	Rabbit: 0.125 mg NHP: 2 mg *	0, 2, 4 wk C (NHP): 8 wk	Yes	Th1-biased response Increased vaccine potency could be due to NFIS
COVID-19 [55]	VIU-1005 (Spike)	Mouse	Tropis ^†^ (ID, IM)	0.025 mg, 0.05 mg, 0.1 mg	0, 2, 4 wk	Yes	Th1-biased response NS ineffective NFIS dose-sparing and less variable responses Dose-dependent response
COVID-19 [87]	pVAX-S1 (Spike S1)	Mouse	Tropis ^†^ (IM)	0.025 mg, 0.05 mg	0, 3, 6 wk	No	Th1-biased response No difference between doses
Influenza [16]	VRC-FLUDNA082-00-VP (HA)	Human Ph 1	Stratis (IM)	4 mg	0, 16 wk	No	Heterologous prime–boost with ferritin NP vaccine Heterologous regimen more immunogenic Durable and cross-reactive responses
Influenza [81]	HA	NHP	Tropis (ID)	0.075 mg	0, 6, 12 wk C: 16 wk	No	APC-targeted vaccine NFIS is “pain-free”
HIV [88]	DNAGC5 (Env, Gag)	Rabbit	Stratis (IM)	0.2 mg	0, 4, 8, 12, 20, 28 wk	No	Heterologous prime–boost with MVA and protein vaccines CpG ODN 1826 adjuvant co-injected
HIV [89]	DNAGC5 (Env, Gag)	Rabbit	Stratis (IM)	0.2 mg	0, 4, 8, 12, 16, 20 wk	No	Heterologous prime–boost with poxviral vectors
HIV ^‡^	DNAGC5 (Env, Gag)	Rabbit	Stratis (IM)	0.2 mg	0, 4, 8, 12 wk	Yes	Heterologous prime–boost with MVA vector vaccine NFIS improved antibody responses
HIV [90]	SIV-gag (Gag)	NHP	Stratis (IM)	1 mg	0, 28, 56, 84, 211 d	No	Shared and unique CD8+ TCR in vaccinated NHP as SIV-infected

* = total dose administered in multiple injections at the same timepoint; † = modified Tropis used; ‡ = manuscript in preparation; NFIS = needle-free injection systems; vs. = versus; NS = needle and syringe; COVID-19 = coronavirus disease 2019; ID = intradermal; wk = week; NHP = non-human primate; C = challenge; Ph = phase; VOC = variant of concern; IM = intramuscular; Th = T helper; HA = hemagglutinin; NP = nanoparticle; APC = antigen-presenting cell; HIV = human immunodeficiency virus; MVA = modified vaccinia Ankara; ODN = oligodeoxynucleotide; SIV = simian immunodeficiency virus; d = day; TCR = T-cell receptor.

**Table 3:** Emerging infectious disease plasmid DNA vaccines administered with NFIS.

Indication	Name (Immunogen)	Species	Device (Route)	Dose	Schedule	Vs NS	NFIS Notes
VEEV [94]	pWRG/VEE (E3-E2-6K-E1)	NHP	Tropis (ID) Stratis (IM)	0.4 mg, 2 mg	0, 4 wk	No	IM route more immunogenic while ID more protective
Hantavirus (SNV, ANDV) [95]	M	Rabbit, NHP	Stratis (IM) IMv1 IDv1	Rabbit 0.4 mg, 2 mg, 4 mg * NHP 1 mg *, 2 mg *	0, 4, 8 wk	Yes	NFIS more immunogenic than NS; IM more than ID Combining vaccines feasible NFIS more pragmatic, less cumbersome than EP and PMED
Hantavirus (ANDV) Zika [96]	ANDV: M ZIKV: PrM, E	Rabbit, NHP	Stratis (IM)	Rabbit LNP: 0.001–1 mg No LNP: 0.1 mg NHP LNP: 0.1 mg, 0.3 mg No LNP: 0.1 mg, 0.3 mg, 2 mg	Rabbit 0, 27 d 0, 42 d NHP 0, 28, 56 d	No	Used Arcturus Therapeutics LUNAR LNP formulation In vivo, LNP formulation increased immunogenicity and showed dose sparing In vitro, LNP formulation showed increased protein expression
Zika [97]	VRC5283, VRC5288 (PrM, E)	NHP	Stratis (IM)	1 mg, 4 mg	0, 4 wk C: 8 wk	No	Correlation between neutralizing response and protection
Zika [98]	VRC5283 (PrM, E)	NHP	Stratis (IM)	1 mg	0, 4 wk C: 30, 60, 90 d	No	Pregnant macaque model Durable nAb response that correlated to aviremia Transplacental antibody transfer and improved fetal outcomes
Zika [17]	VRC5283 (PrM, E)	Human Ph 1	Stratis (IM)	4 mg *	0, 4, 8 wk	Yes	NFIS 100% seroconversion, higher immunogenicity than NS Split dosing yielded higher immunogenicity

* = total dose administered in multiple injections at the same timepoint; NFIS = needle-free injection systems; vs. = versus; NS = needle and syringe; VEEV = Venezuelan equine encephalitis virus; NHP = non-human primate; ID = intradermal; IM = intramuscular; wk = week; SNV = Sin Nombre virus; ANDV = Andes orthohantavirus; M = membrane; IMv1 = first-generation PharmaJet IM NFIS; IDv1 = first-generation PharmaJet ID NFIS; EP = electroporation; PMED = particle-mediated epidermal delivery; ZIKV = Zika virus; PrM = pre-membrane; E = envelope; LNP = lipid nanoparticle; d = day; C = challenge, nAb = neutralizing antibody; Ph = phase.

## Data Availability

Not applicable.
